# Supplementary material for: Are carbon emissions trading and green financial instruments synergistic? -Comprehensive quantitative research based on content analysis
Source: PLoS One. 2024 Mar 7;19(3):e0298601. doi: 10.1371/journal.pone.0298601 (PMC10919601; doi:10.1371/journal.pone.0298601)
Supplement: S1 Data — (ZIP) [file pone.0298601.s001.zip › Data/地级市Z_F工作报告.zip!/╔╜╬≈/╤⌠╚¬╩╨/└┤╫╘╣1⁄2╓┌║┼í╛┬φ┐╦╩2╛▌═°í┐_2014.docx]

各位代表：

现在，我代表区人民政府向大会报告工作，请予审议，并请各位政协委员和其他列席人员提出意见。

一、2013年工作回顾

2013年，是全区经济社会发展不平凡的一年。一年来，在区委的正确领导下，在区人大的法律监督和区政协的大力支持下，区政府团结带领全区人民，以科学发展观为指导，砥砺奋进，负重前行，圆满完成年初确定的主要目标任务。全区呈现出经济建设稳中有进、社会事业稳中有为、各项工作稳中向好的发展态势。

国民经济持续较快增长。2013年，全区生产总值完成78.3亿元，增长11.2%；财政总收入完成9.98亿元，公共财政预算收入完成5.2亿元，增长1.9%；规模以上工业增加值完成27.1亿元，增长15.4%；固定资产投资完成70.7亿元，增长26%；社会消费品零售总额完成12.9亿元，增长10.3%；农民人均纯收入达到10289元，增长11.8%；城镇居民人均可支配收入达到19360元，增长10.3%。

重点工程项目有序推进。认真落实“六位一体”工作机制，全年引进百万元以上经济技术合作项目66项，实际到位资金75.8亿元；实施投资项目100个，完工41个，完成投资45.8亿元，以河坡电厂“上大压小”、华佳发煤站改扩建为重点的“336”工程项目进展顺利。307复线小微企业集聚区建设初见成效，阳煤集团龙兴腾森铸造等7个项目相继入驻。保安煤业150万吨项目正式投产，鸿泰煤业45万吨项目转产验收，旧街、神堂和坡头等标准化矿井建设按进度实施。

农业农村工作扎实有效。全年生产粮食2990万公斤，生猪存栏达到54500头，蛋鸡存栏200万只，奶牛存栏1032头，新增温室大棚200亩、果园1300亩、养殖小区10个、省级“一村一品”专业村11个。裕盛源调味品生产线建成投产，宝鑫养殖场建设顺利推进。成功举办了桃林沟第十届“桃花节”、第四届“关公文化旅游节”和西南舁首届“苹果采摘节”，一批乡村旅游项目悄然兴起。184个行政村清洁工程全面启动，81个行政村街道亮化全部完工，新增的15个省级新农村建设重点推进村“四化四改”和“五个一”工程全面完成，平坦镇芦湖等4村顺利搬迁。

人居环境质量明显提升。坚持打好荫营城区环境集中整治和高速公路两侧生态修复两场硬仗。荫营城区“五网”全部入地，供水、供热管网改造顺利完工，大型运输停车场建设有序推进；京昆高速17.7公里通道绿化全面完成，全区新增造林面积3万余亩，森林覆盖率达到25.8%。积极配合生态新城重点工程建设，漾泉大道一期进入扫尾阶段，保晋路拆迁如期完成，珍宝园建设、市检察院技侦中心大楼等工程进展顺利。大气污染防治有效加强，“4+2”约束性指标严格控制在市下达指标范围之内，荫营城区二级以上天气达到337天，荫营镇跨入“省级生态乡镇”行列。

转型综改试验迈出新步。编制完成《2013-2015年综改实施方案》和《2013年行动计划》。大力实施“六创六破”破解发展难题：制定出台土地“八统一”管理办法，报批各类建设用地3500余亩；实施“助保金贷款”，帮助8家中小企业获贷3050万元。扎实推进扩权强区试点，六大类43项下放扩权事项有效承接。创造性地开展以照“镜子”、扬“鞭子”、给“票子”、丢“面子”、摘“帽子”为主要内容的“五子登科”考核，政府各级干部干事创业的主动性和积极性显著增强。

民主法治建设不断加强。认真贯彻执行区人大及其常委会的决议、决定，积极支持区政协履行职能，自觉接受区人大的法律监督和区政协的民主监督，全年办理人大代表意见、建议101件，政协委员提案169件。扎实开展“六五”普法教育，深入推进“法治郊区”建设，严厉打击各类违法犯罪，人民群众的安全感、满意度进一步提升。桃林沟村获“全国民主法治示范村”称号。

安全生产形势总体平稳。认真落实安全生产政府监管责任和企业主体责任，扎实开展煤矿、非煤矿山、建筑施工、人员密集场所、消防安全、道路交通、危险化学品、民爆物品、锅炉压力容器、特种设备、燃气管道以及校园安全、食品安全、护林防火、防汛防灾等行业和领域安全大检查，各项安全指标有效控制在市政府下达指标之内，煤矿百万吨死亡率连续5年为零。始终保持严厉打击私挖滥采的高压态势，全年共关闭取缔非法采矿坑点185处，立案查处12起，行政拘留5人，刑事拘留18人，判刑2人，处分党员干部9人。

社会各项事业全面进步。年初确定的“幼教资源优化配置和农村卫生医疗提质”两件实事全部兑现。财政拿出175万元为487名村办幼儿教师每人每月补助300元，新招聘的40名幼儿教师和50名小学教师全部上岗，桃林沟等5所标准化幼儿园建设进展顺利；实现了60岁以下村医养老保险全覆盖，新选聘的40名大学生村医充实到基层一线，为81个村级卫生所配备了血糖仪、制氧机等医疗设备，区急救中心和李家庄、杨家庄卫生院改造工程圆满完成。公立医院改革全面启动，区人民医院与北京301医院开通了远程诊疗系统，新农合“一卡通”和参合农民住院免缴押金等惠民措施有效落实。就业和社会保障体系进一步完善。全年新增城镇就业岗位4010个，转移农村剩余劳动力2812人，建成保障性住房808套，完成农村危房改造100户，荫营敬老院获“全国文明敬老院”称号，“暖心煤”发放在8月底提前完成。计划生育“为生命护航”行动深入开展，出生人口素质不断提高。全年申请科技专利240余件，10家企业成为省、市专利工作试点。妇幼、档案、文化工作再创佳绩，我区成为全省妇女儿童“实施两纲两规示范县区”；区档案馆在全省率先晋升为国家一级档案馆；河底镇被评为全省第二届文化先进乡镇，李家庄乡和区文体广电局获全省农家书屋建设先进集体称号。二轮修志工作圆满完成，《阳泉日报•郊区周刊》、阳泉电视台《直通郊区》栏目相继刊播。粮食、供销、双拥、武装、宗教、防震减灾等各项工作都取得新进展。

各位代表，一年来，面对经济下行压力，我们不畏艰险、迎难而上，在复杂、困难的形势下，取得了令人鼓舞的业绩。这些成绩的取得，是区委正确领导的结果，是区人大有效监督的结果，是区政协大力支持的结果，更是全区人民齐心协力、真抓实干的结果。在此，我代表区政府，向为郊区发展付出辛勤劳动、做出无私奉献的广大干部群众致以亲切的问候和崇高的敬意！向所有关心、支持和帮助郊区发展的各位人大代表、政协委员、社会各界人士表示衷心的感谢！

在肯定成绩的同时，我们也必须清醒地看到，全区经济社会发展中还存在一些不容忽视的问题：传统产业提升不快，新兴产业培育不足，招商引资成效欠佳，转型发展任重道远。农村集体经济弱化，农民增收渠道不宽，基础设施建设滞后，生态环境欠帐较多。特别是安全生产基础还不牢固，打击私挖滥采任务艰巨，社会管理难度日益加大，平坦镇“6.1”农用车载人翻车事故、区法院“10.22”审判大楼工地坍塌事故、大阳泉“12.18”盗采国家资源案件等事件的发生，不仅给人民群众的生命财产造成了损失，而且在社会上也形成了一定的负面影响。对于这些问题，我们必须高度重视，强化措施，认认真真下大力解决！

二、2014年工作安排

2014年，是全面贯彻落实党的十八届三中全会精神、深化改革的开局之年，是实施“十二五”规划、加快综改试验区建设的关键之年。根据区委八届四次全体（扩大）会议和全区三级干部会议精神，今年全区经济社会发展的指导思想是：以邓小平理论、“三个代表”重要思想、科学发展观为指导，全面贯彻党的十八大和十八届三中全会精神，按照稳中求进、改革创新的总要求，抢抓转型综改、扩权强区试点和生态新城建设的重大机遇，全面实施产业强区、拓城靓区、综改活区、民生安区、实干兴区“五大”战略，完成八大指标，实施八大项目，建设八大工程，办好八件实事，抓好六项工作，为打造生态宜居新城、建设现代城郊强区而努力奋斗！

完成八大指标：

——全区生产总值增长10%，达到86亿元；

——公共财政预算收入增长8.4%，达到5.64亿元；

——规模以上工业增加值增长14%，达到28.5亿元；

——全社会固定资产投资增长23%，达到87亿元；

——城镇居民人均可支配收入增长10%，达到21300元；农民人均纯收入增长10%，达到11320元；

——社会消费品零售总额增长10%，达到14.2亿元；

——万元GDP综合能耗下降3.5%；环保“4+2”约束性指标控制在市政府下达指标范围之内；荫营城区二级以上天气保持在330天以上；

——人口自然增长率控制在4‰以内；安全生产指标控制在市政府下达指标范围之内。

实施八大项目：一是继续实施河坡电厂2×35万千瓦热电联产项目，开工建设2×1.5万千瓦垃圾焚烧发电项目；二是完成神堂煤矿90万吨、旧街煤矿60万吨和坡头煤矿45万吨标准化矿井建设项目，开工建设保安煤业2.8万千瓦瓦斯发电项目；三是开工建设中庸年产40万吨陶粒砂项目一期工程；四是开工建设山西新盟纸业年产12万吨石头造纸项目；五是完成宝鑫现代养殖场年出栏2万头生猪项目，开工建设阳泉市农产品批发交易市场；六是实施桃林沟鑫利达游乐园项目；七是启动建设阳泉市汽车文化综合体项目；八是启动新梦想生物工程有限公司微生物制剂及粗纤维复合型饲料项目。

建设八大工程：一是实施荫营环城绿化和刘备山生态修复工程；二是完成漾泉大道一期工程，开工建设二期工程；三是完成珍宝园和南区新城雅馨园建设工程，开工建设恒大帝景一期工程；四是开工建设东城科技大道，完成中兴大道建设工程；五是启动三泉、魏家峪、大西庄保障性住房建设工程；六是继续推进阳泉职业技术学院新校区和市规划展览馆建设工程；七是配合实施阳大铁路郊区段28公里建设工程；八是完成太阳高速河底出口连接线工程。

办好八件实事：一是启动行政村通公交全覆盖工程，切实解决老百姓出行难的问题。二是实施荫营城区供水管网改造及水质净化工程，切实解决城区居民饮水质量不高的问题。三是实施边远山村任职教师岗位津贴全覆盖工程，鼓励优秀教师到山区任教。四是实施参合农民大病商业保险制度，村卫生所就诊免收一般诊疗费，低保对象免费享受新农合医保，完善乡村医生养老保险制度，切实解决农民看病贵和老年村医生活保障的问题。五是启动区社会福利综合服务中心建设，切实解决“五保”老人、优抚对象、孤儿、流浪乞讨人员等弱势人群的抚养问题。六是深入开展“助保金贷款”业务和土地收益保证贷款试点工作，切实解决中小微企业和农民融资难、融资贵的问题。七是进一步抓好乡村清洁工程的实施，切实解决农村环境脏乱差的问题。八是实施农民技术培训教育工程，切实增强农民致富增收的能力。

抓好六项工作：

（一）坚持双加双培不动摇，突出外引内聚，着力加快产业转型升级

郊区的希望在转型，转型的关键在产业，产业的根本在项目。推进产业强区，必须大力实施“煤耐+新型”、“园区+项目”的“双加双培”工程，打牢发展基础，推进产业再造。

围绕“煤耐+新型”，培育六大主体产业。按照“稳定煤炭、提升耐火、巩固建材、强推电力、发展制造、做精旅游”的思路，积极构建多元发展的产业体系。

——稳定煤炭。一是力促旧街煤矿60万吨、神堂煤矿90万吨、坡头煤矿45万吨三大基建矿井分别在6月底、10月底、年底前投产运行，确保煤炭产量达到280万吨以上。同时，支持南煤集团西上庄500万吨新建矿井早日开工。二是继续实施煤炭企业“1+1”工程，开工建设保安煤业2.8万千瓦瓦斯发电项目，推进燕煤集团输送机项目建设。三是大力支持燕煤集团300万吨洗煤、华佳发煤站尽快达产，完成正舜180万吨洗煤生产线项目，提高煤炭转化增值率。

——提升耐火。一是加强资源管控。依托世界500强中铝集团等大型企业对资源进行整合重组，搭建区域性耐火原料和产品集散交易中心，积极探索资源管理和生态保护双赢的可持续发展之路。特别是要下大力取缔非法违法矾石土竖窑，大力推广采用洁净能源烧成的新型窑炉，提高资源利用率。二是实施外引内联。支持金隅通达以及千亨、华岭等区内有实力的耐火企业规模化发展，采取市场引导、政府搭桥、政策促动等措施，鼓励企业以股权交易等方式走联合壮大、合作共赢之路。三是创新营销模式。以德旭“真金”耐火材料交易网为依托，加快电子商务平台建设，切实推动耐火产业向新型化、信息化、网络化迈进，力争年内实现网上交易100亿元以上。四是推进技术升级。充分发挥我区国家级硅铝质耐火材料质检中心、耐火材料研究所以及耐火协会的作用，加快业内科研技术力量整合，整体提升行业核心竞争力。今年，区政府要制定出台《促进耐火产业健康发展的实施意见》，挂靠经信局成立铝耐管理中心，对郊区耐火的整合、重组、提升、管理等方面进行统筹协调和服务。同时，筹措各类政策性资金1000万元以上，在耐火企业改造升级、设备更新、联合重组、产品研发等方面给予扶持。

——巩固建材。支持冀东水泥巩固已有市场，提高产量，扩大营销半径，力促低温余热发电项目尽快投产运行；扶持天隆工程材料公司加快产品开发和技改提升；鼓励煤矸石砖、粉煤灰砖等新型建材扩大生产规模，助推天华20万吨净水剂、众辉30万吨超细粉以及益汇、百通、万方混凝土等项目投产达效。

——强推电力。支持河坡电厂2×35万千瓦热电联产机组项目加快建设进度，开工建设2×1.5万千瓦垃圾焚烧发电项目。认真做好西上庄煤电一体化2×60万千瓦发电机组项目的前期准备，力争使电力工业成为我区新的支柱产业。

——发展制造。支持广凯机械制造技改提升、丰裕矿山机械矿用设备维修、神华重泰仪器仪表制造、盛德矿用设备生产线和立博线缆电缆技改等项目实施，助推华鑫电器、华越机械增产增效，着力引进湖南华骏5000台减速器项目，尽快做大做强装备制造业。同时，加强与市水泵厂、阀门厂对接协调，力促有关搬迁扩建项目落户我区。

——做精旅游。科学规划，精心策划，合理定位，加大宣传，大力开展“乡村旅游年”活动，切实推动郊区乡村旅游发展。按照制定出台的《关于发展乡村旅游的实施方案》，今年，重点要打造桃林沟生态娱乐园、西南舁休闲采摘园、刘关张忠义文化园、辛庄红色旅游园、小河历史民俗园、三明酒庄文化园等六大乡村旅游基地，建设新城森林公园、保安生态沟、龙泉沟垂钓园等三大观光园区，举办桃林沟桃花节、关王庙关公文化节、翠枫山红叶节和冰雪节等十大旅游项目推介活动，真正通过以景促游、以游聚客、以节造势，实现乡村旅游与文化产业的融合发展，农民增收与农村繁荣的互利共赢。

围绕“园区+项目”，培育新的经济增长极。充分发挥园区经济的集聚效应，以平台建设为依托，以招商引资为路径，以项目建设为载体，全面加快产业结构调整。

——打造园区平台。按照“政府策划、乡镇组织、企业实施、社会参与、多元共建、分步推进”的原则，创新建设资金和利益分享机制，致力引进阳煤集团、南煤集团、燕煤集团、荫营煤矿、固庄煤矿、煤运公司等国有大集团、大企业参与园区建设。区政府将制定出台《平台建设实施方案》，以乡镇为单位，根据全区平台建设总体规划、乡镇地域特色、产业发展需求，严格按照省、市建设标准，精心打造，务求实效。今年，要率先开发“一园一区”。“一园”即东城科技创新园，重点是开工建设总投资2.9亿元的东城科技大道，依托阳煤集团装备制造园和开发区信息产业园项目，力争形成水、电、暖、气、路、通信“六通一平”的项目平台4000亩以上。“一区”即河底新兴工业集聚区，要在完成建设规划的基础上，依托固庄煤矿和区煤运公司，采取平台整体招商的方式，力争形成建设净地1000亩以上。同时，继续推进307复线小微企业集聚区的平台建设，启动双营路汽车贸易物流园建设。

——加快招商引资。要修订完善优惠政策和办法，制定出台《加快招商引资的实施方案》。一是强化业务部门尤其是部门领导在招商引资工作中的示范作用，大力开展“领导干部赴一线、抓招商、促发展”活动，以部门招商促进领导招商，带动企业招商，形成全民招商。二是充分发挥驻区大企业的产业延伸、辐射带动和人脉优势，积极邀请国家部委、大集团、大企业来我区考察，不断加大区内优势项目对外推介力度。今年，要组织好两个以上招商引资推介会，力争在项目引进上有新突破。三是充分利用现有园区平台，在科学定位产业功能、提供多样化合作方式的基础上，采取整体打包或切块包装的办法，大力实施园区招商。四是大力发展“飞地经济”，切实加强与城区、矿区、开发区的互动合作，加快飞地经济项目的引进、服务和建设，真正使合作项目“飞得进、落得下、建得起、有实效”。

——狠抓项目实施。今年，我区计划实施投资项目102项，总投资336.8亿元，当年计划投资90.6亿元。其中，重中之重是“八大项目”和“八大工程”。为确保项目建设顺利推进，今年，区政府将成立三大中心：一是挂靠发改局成立项目储备中心，采取为储备项目提前办立项、报可研的办法，切实解决项目手续办理难、推进慢的问题。二是挂靠招商局成立项目招商中心，白泉工业园区管委会、经信局、工商联等部门共同参与，切实解决招商引资渠道单一、力量不足的问题。三是挂靠监察局成立项目服务中心，发改、经信、招商、规划、国土、环保等有关部门参与，创优投资环境，提高办事效率，切实解决项目服务不优的问题。

各位代表，郊区人多地少、资源枯竭的实际，决定了转型发展是我们实现富民强区的唯一出路。尽管任重道远，但只要我们认准目标，不断积小胜为大胜，积跬步以千里，就一定能走出一条有效益、有质量、可持续发展的转型之路！（二）坚持农民增收不动摇，做精做优一产，大力发展现代城郊农业

要充分发挥我区环城而立的先天优势，紧紧瞄准阳泉市区50万人的餐桌消费，打好特色牌，走好增收路。

做强菜、果、蛋三大主导产业。一是建设以蔬菜为主的河底现代农业综合园，年内新增温室大棚200亩、标准化蔬菜基地500亩。二是打造以苹果为主的西南舁万亩优质果品园，年内新增苹果种植1000亩以上、标准化果园500亩以上。三是做大以蛋鸡为主的平坦•义井双百万蛋鸡养殖园，扩大桃林沟金凤凰、泊里养殖场规模，抓好大村瑞峰养殖场、坡头绿源蛋鸡养殖场扩建，年内新增蛋鸡存栏30万只以上。同时，要围绕市民休闲观光，抓好桃林沟现代综合农业园、汉河沟农产品加工采摘园以及上千亩坪、小河、咀子上、南沟等农业观光园建设，开辟农民增收新途径。今年，区政府要制定出台《关于加快“菜篮子”工程建设、推进城郊农业发展的实施意见》，拿出1000万元支持农业发展。

加快农业产业化进程。一要加快经营机制转变，积极尝试“合作社+基地+品牌”发展模式，加大农超对接、农产品定点直销、配送专卖等经营模式推广力度，年内新增农超对接示范基地500亩。二要充分发挥北方科技、易汇投资、三来食品、裕盛源等龙头的辐射带动作用，加快宝鑫现代养殖场建设，叫响“三舁”苹果、“冬春”蔬菜、“晋阳府”食醋、“老妮儿”食品等特色品牌，助推农业走标准化生产、品牌化经营之路。三要继续加大“一村一品”专业村创建力度，力争年内省级专业村总数达到40个以上。

积极推进土地流转。一是向大户集中。采取支持家庭农场发展、鼓励联户规模经营等有效办法，有序推进土地资源向种植业、养殖业大户流转。今年，区政府要对土地流出户每亩一次性补贴100元。二是对市民开放。要围绕知青对农村的怀旧、城市中老年人对土地的热爱、青少年对农事的好奇和兴趣，鼓励有条件的村建设“知青大院”、开办“市民农庄”、创办“花卉乐园”，发展土地租养、大棚领养、花卉托养等特色服务。三是让企业开发。充分利用山庄窝铺移民搬迁后的耕地、林地、宅基地资源，采取政府给政策、企业拿资金、农民出劳力的办法，实现闲置土地的产业性开发，促进农民增收。

大力实施“百企千村”产业扶贫工程。建立健全村企直通、定期互动机制，坚持主动出击、积极作为，不断加强与南煤集团、燕煤集团、河坡电厂、荫营煤矿、固庄煤矿、煤运公司、志远工程公司等企业的合作，真正通过发展产业给贫困山区农民授之以渔。今年，重点要抓好保安沟万亩生态林、南沟生态休闲观光园、刘备山休闲观光园、燕龛万头猪场扩建、西南舁万亩果园改造提升和休闲采摘园、五里庄2万只育肥羊养殖园、神堂沟休闲采摘观光农业园、下烟村200亩集中连片农业园、杏树坡10万只蛋鸡养殖园等项目对接，力争早落地、早开工、早见效。同时，认真抓好农民培训尤其是职业农民培训，力争年内完成2万人次以上。在旧街乡积极开展农村土地承包经营权确权发证试点工作，切实保障农民权益，赋予他们更多的财产性权利。

进一步改善农村落后环境面貌。一是投资1500万元启动行政村通公交全覆盖工程，解决农民出行难的问题。二是投资450万元继续实施乡村清洁全覆盖工程，解决农村环境脏乱差的问题。三是投资1200万元实施矾窑等14村环境连片整治工程，切实改善荫营城区周边村环境质量。四是投资200万元完善农村饮水安全工程，再解决5000人的饮水安全问题。五是认真组织两轮“五个全覆盖”回头看，深化147个重点推进村的“四化四改”和“五个一”工程，建立健全长效管护机制。六是加强温河灌区运行管护，严守耕地保护红线，不断提升农业综合生产能力。

各位代表，农业基础弱、农村环境差、农民增收难，仍然是制约我们打造生态宜居新城、建设现代城郊强区的最大短板。我们一定要按照“多予、少取、放活”的方针，持之以恒地确保农业投入只增不减、扶持力度逐年加大、扶贫攻坚常抓不懈，真正让农业火起来、农村活起来、农民富起来！

（三）坚持基础先行不动摇，加快扩容提质，倾力打造生态宜居郊区

要紧紧抓住市委、市政府“扩城阔市”的战略契机，坚持城乡互动、一体发展，大力推进以人为核心的新型城镇化。

主动融入生态新城建设。要充分发挥我区生态新城建设的主场优势，主动对接、主动配合、主动作为，真正做到在前置服务上周到细致、项目建设上配合有力、内外规划上步调统一、利益分配上互利共赢。一是围绕3平方公里生态新城起步区建设，完成漾泉大道一期工程，配合实施好漾泉大道二期、平阳路一期、阳泉至大寨铁路郊区段、三泉村整体搬迁、市职业技术学院、市规划展览馆、市中心人民医院、市养老公益苑等重点工程建设。二是按照“拆小村、建大区”的原则，整体谋划生态新城范围涉及村的搬迁安置和集中建设。同时，结合有关政策，积极探索搬迁村“村变居”、搬迁户“农转非”等城镇化推进新路子。三是认真落实拆迁补偿、就业安置、失地农民养老保险等措施，探索村集体和农民个人入股参与新城建设的新机制，确保农民失地不失业、失地不失利。

加快荫营城区扩容提质。按照融入阳泉生态新城的建设标准，高起点规划、高品位设计、高质量建设，全力打造郊区人自己的“城市名片”。一是放大发展空间，将荫营城区面积按新的规划逐步扩大到8平方公里以上，完善行政、商贸、文教、居住、物流、生态等功能区划分，力争用三到五年时间，将荫营建设成为设施完善、低碳绿色、环境优美的风情小城。二是提升发展品位。抓好荫营东西大街、外环路以及江正大街的综合整治和美化绿化，积极在街道沿线、主要节点规划一批宜景则景、宜园则园的小游园，努力打造优雅宜人的阳泉“后花园”。三是完善城市功能。加快南区中兴路和大型运输车辆停车场建设进度，推进城区雨污分流管网改造和荫营河治理。特别是要大力实施荫营城区水质净化工程，真正让城区百姓喝上洁净、安全、放心的饮用水。四是提高管理水平。切实加大沿街广告、装饰以及车辆乱停乱放和马路市场的整治力度，确保城区环境整洁有序。同时，加快部门和单位专业网络整合，打造数字化城市管理平台，启动“智慧郊区”建设。

推进中心集镇改造提升。河底镇要充分发挥阳五、京昆两条高速过境的交通优势，依托“百镇工程”和平台建设，加快人口和产业集聚，建设新型工业重镇。平坦镇要充分利用阳煤驻地的地缘优势，加强地企合作，加快山庄窝铺的移民搬迁，推动区域经济协调均衡发展。义井镇要按照城市功能区定位，打破所属行政村地域界线，统筹规划、分步推进，努力打造阳泉的居住休闲区和三产服务区。李家庄乡、杨家庄乡要按照生态新城建设总体规划，主动介入，积极作为，配合好各项重点工程项目的实施。西南舁乡、旧街乡要因地制宜，科学规划，加强基础设施建设，加快人口商贸集聚，着力在特色集镇建设上迈出新步伐。

大力发展三产服务业。一是加快李荫路、双营路、漾泉大道等道路两侧开发，大力扶持东城钢材市场、冀东物流园、通宝鑫能物流园建设，积极引进华港新能源重卡物流园等项目，切实加快物流业发展。二是抓好山西致诚汽贸、鼎盛汽贸等4S店建设，做好河北邯郸远洋集团汽车文化综合体项目前期准备，逐步扩大汽车贸易。三是规划建设荫营商贸服务功能区，加快电子商务、网络业务、金融服务、时尚消费、楼宇经济等新型服务业培育。同时，抓好专业市场建设，支持有条件的乡镇和村发展底商经济，开展家政服务，增加居民收入。

加强生态环境整治。一是大搞植树造林。重点抓好阳五高速30公里生态修复、西外环高速12公里通道绿化和刘备山森林公园8公里绿色长廊建设。二是加强地质灾害治理。用足用好国家和省、市采煤沉陷区治理各项政策，加强与阳煤等相关企业的对接协调，切实完成好平坦镇南山、石板片等5村的移民搬迁。三是推进减排治污。加大天然气、煤层气等清洁能源供应推广力度，扩大集中供热实施范围，从严控制二氧化硫、烟（粉）尘等主要大气污染物排放，确保荫营城区二级以上天气保持在330天以上，空气质量稳定达到国家二级标准。

各位代表，建设功能齐全的“荫营城”，打造生态宜居的新郊区，不仅是我们加快城镇化的必由之路，更是广大人民群众追求美好生活的殷切期盼。我们一定要抢抓生态新城建设带来的难得机遇，顺势而为，乘势而上，真正把郊区建设成为富有生机、充满活力、独具魅力的美丽家园！

（四）坚持深化改革不动摇，创新体制机制，努力增强发展内生动力

今年，是省委、省政府确定的“转型综改攻坚年”。作为转型综改和扩权强区的双重试点，我们一定要狠抓政策机遇，大胆先行先试，推进转型跨越。

创新民营经济发展。从先进地区发展经验来看，民营经济是最具活力的增长因素，也是最有潜力的发展主体。推进转型跨越，必须千方百计加快民营经济发展。要认真落实营改增、小微企业税费减免等有关政策，助推民营经济减负增收。要用足用好上级关于鼓励民营企业、民间资本参与转型综改重大项目的扶持政策，积极建设面向民间资本开放的投资项目库，确保民营企业与国有企业、招商引资企业在同等条件下公平享受政策、公平参与竞争、公平获取发展要素。今年，省政府提出在全省培育年销售收入超亿元的民营企业100户以上，我区要重点扶持天隆工程材料和天华净水剂组建集团公司，力争实现民营“小巨人”企业零的突破。

创新金融融资服务。一是继续推行“助保金贷款”业务，再拿出1000万元政府风险补偿金，使助保金贷款融资规模达到2亿元以上，力争将更多的企业纳入“重点企业池”。二是积极开展土地收益保证贷款试点工作，大力推行用土地承包经营权流转预期收益作为贷款抵押的融资新模式，切实解决农民融资难、融资贵的问题。三是充分发挥金融办、小额贷款公司等现有金融机构的作用，广泛吸纳社会闲散资金，集中小钱办大事。四是借鉴襄垣、灵石经验，大力扶持民间借贷市场发展，拓宽民间资本投融资渠道，切实为重大转型项目提供强有力的资金保障。

创新项目用地管理。一是要完善土地储备、用地供应、土地交易、地价管理、推介招商、出让管理、征地拆迁、集中安置等“八统一”土地管理办法，力促现行土地管理机制更加适应全区经济社会发展的需要。二是要充分发挥土地储备中心作用，尝试提前为园区平台办理土地利用规划、土地占用指标等相关手续，最大限度确保招商引资项目建设用地。同时，继续推进增减挂钩，确保占补平衡。三是要积极探索农村集体经营性建设用地出让、租赁、入股以及与国有土地同等入市、同权同价的新机制，切实搞好试点申请工作，力争抓住政策机遇先行一步。四是要加强对占地不落地、拿地不投入、圈地不建厂、承诺不兑现以及擅自变更土地用途招商项目的清理和规范，采取多种方式迁建、拆并、整合现有企业用地，通过“腾笼换鸟”增加项目建设用地。

深化行政管理体制改革。认真抓好“扩权强区”下放事项承接工作，严格执行“两集中、两到位”要求，确保应进入行政审批大厅的服务单位在6月底前全部进驻。大力实施建设项目联合预审制度，变串联审批为并联审批，真正做到减环节、压时限、强服务，创优政务环境，提高办事效率。积极稳妥推进事业单位机构改革，确保财政供养人员只减不增。

（五）坚持民生为先不动摇，狠抓增量提质，致力均衡普惠再续新篇

把握民生脉动，关注民生诉求，解决民生问题，是见证和检验政府执政能力的重要标尺。我们一定要不断加大民生领域投入，加快民生事业发展，让百姓生活得更加幸福、更有尊严。

努力办好人民满意的教育。全力推进义务教育发展基本均衡县（区）建设，确保年内通过国家级验收。大力实施边远山村任职教师岗位津贴全覆盖工程，由区财政出资，按学校距离市区远近分类分别每月补助150元和100元，切实鼓励优秀教师到山区任教，推进教育公平。继续引深幼教资源优化配置工程，在去年为村办幼儿教师每月补助300元的基础上再加300元。进一步改善办学条件，完成好乡镇中心幼儿园建设全覆盖工程和中小学（幼儿园）安装净化水设备全覆盖工程。

继续推进农村卫生医疗服务提质。重点实施参合农民大病商业保险制度。今年，区财政要拿出180万元，为参合农民购买商业保险，对大额医疗费用新农合补偿后的个人自付部分，通过商业保险再给予55%-80%的二次补偿，切实解决农民“因病致贫、因病返贫”的问题。同时，对参合农民实行村级卫生所免收一般诊疗费，让低保对象免费享受新农合医保；完善乡村医生养老保险制度，建立健全村医退养机制；深化公立医院改革，落实零差价销售、单病种限费、服务价格调整等惠民政策；完善区、乡、村医疗卫生机构一体化管理运行机制，推进新农合规范化、制度化运行，进一步扩大受益面，提高受益度。

加快就业和社会保障体系建设。认真落实就业创业扶持政策，多渠道开发就业岗位，建立和完善多元化就业培训体系，加大劳务输出、就业服务工作力度，做好高校毕业生、就业困难人员、退伍军人等重点人群就业工作，消除“零就业”家庭。严厉打击非法用工，大力实施农民工工资保证金制度，坚决杜绝拖欠农民工工资行为。进一步推进养老、医疗、失业、工伤和生育等“五大”保险提质扩面，加强特困家庭和残疾人救助，加快保障性住房建设，抓好农村养老照料中心试点工作，启动区社会福利综合服务中心建设，提高社会保障水平。

推动社会各项事业全面进步。大力倡导社会主义核心价值体系，不断加强公民道德和精神文明建设，努力在全社会营造知荣辱、讲正气、树新风、促和谐的良好风尚。要制定《郊区文化产业中长期发展规划》，设立繁荣文化艺术专项资金，认真组织建区三十年“文化惠民、欢乐基层”下乡慰问演出活动，切实丰富群众文化生活。增加科技投入，加快科技创新，力争全年申请专利240件以上。开工建设区“烈士陵园”，推动“双拥”工作再上新台阶。加快档案资源整合，推进数字化档案馆（室）建设。积极启动《郊区年鉴》编写，完成《郊区党史》编纂工作。同时，加强国防教育和后备力量建设，继续推进计划生育、粮食、供销、宗教、妇女儿童、防震减灾等各项事业全面发展。

各位代表，人民对美好生活的向往，是我们的奋斗目标；群众对幸福生活的期盼，是我们的最大职责。我们要按照以人为本、民生为先的根本要求，设身处地为群众着想，尽心竭力为群众办事，诚心实意为群众服务，切实从群众最关心、最需要、最现实的事情做起，让发展的成果泽被千家万户，让改革的红利惠及全区人民！

（六）坚持以人为本不动摇，深化平安创建，全力营造和谐发展氛围

安全是最大的民生，是比泰山还重的责任，是任何时候、任何地方、任何情况都不能触碰的高压红线。我们一定要强化安全发展理念，按照“三个决不能过高估计”的基本判断，时刻以敬畏生命、敬畏责任、敬畏制度之心，扎实做好安全稳定各项工作。

狠抓安全生产不放松。一是坚持“安全第一、预防为主、综合治理”方针，围绕“三杜绝、双下降、一确保”目标，建立健全“党政同责、一岗双责、齐抓共管”的责任体系，不断加大安全考核权重，切实抓好企业主体责任的落实。二是深化煤矿、非煤矿山、建筑施工、人员密集场所、消防安全、道路交通、危险化学品、民爆物品、锅炉压力容器、特种设备、燃气管道以及校园安全、食品安全、护林防火、防汛防灾等重点行业和领域的安全大检查活动，确保安全监管全覆盖、无盲点。三是采取不发通知、不打招呼、不听汇报、不用陪同，直奔一线、直插现场的方式，加大抽查、督查力度。对检查不深入、排查不到位、治理走过场的部门、乡镇和企业，视同发生安全事故严肃处理。四是加强应急管理体系建设。重点抓好护林防火应急分队、国土资源执法分队、公安国土资源犯罪侦查分队、煤矿矿山救护大队和民兵应急分队等五支专业队伍建设，提升突发事件处置和应对能力。今年，区政府将拿出100万元，用于乡镇安全经费专项补贴。

严打私挖滥采不手软。深刻汲取大阳泉“12.18”盗采国家煤炭资源案件教训，充分认识当前私挖滥采的危害性、隐蔽性和复杂性，猛药治疴，重典治乱，对私挖滥采坚决实行“零容忍”。一是完善监管网络体系。落实部门、乡镇和村监管责任，建立定期不定期巡查制度，确保对浅层资源赋存和动力电源接入场所等重点区域、重点部位的有效监管，真正做到经常性地、一户一户地、不留死角地彻底清查。二是严管涉煤涉矿工程。今后，凡新上造地、养殖、修路、新农村建设等项目，必须经主管部门严格审核，区政府批准；对涉及地下资源的土地占用和新上项目，要在严格审批的基础上进行公示，确保有效管理和群众监督。三是加大案件查处力度。对发现的私挖滥采案件，采取国土部门扣押设备、公安部门抓捕涉案人员和打击黑“保护伞”、纪检监察部门开展公职人员失职渎职调查、审计部门对所涉村财务进行审计的办法，多管齐下，重拳出击，严打重处，真正震摄违法犯罪分子。

加强社会管理不懈怠。进一步完善定期接访、联合接访、带案下访等各项制度，大力推广“枫桥经验”，及时发现和解决各类苗头性、倾向性、潜在性问题，切实扭转“信上不信下、信闹不信理、信访不信法”的不良风气。继续深化“平安郊区”、“法治郊区”创建，引深“六五”普法教育，加快“天网工程”建设，提升违法犯罪打击能力。认真做好第十届村民委员会换届选举工作。进一步规范农村“资源、资产、资金”管理，抓好“村财乡管”、“四议两公开”等工作落实，确保农村稳定。

三、全面加强政府自身建设

为政之要，贵在谋事，重在执行。做好今年各项工作，我们必须以党的群众路线教育实践活动为契机，按照习总书记提出的“严以修身、严以用权、严以律己，谋事要实、创业要实、做人要实”的“三严三实”要求，努力建设为民、务实、清廉的责任政府。

要坚持科学施政。一要加强学习。“学者非必为仕，而仕者必为学”。政府各级干部都要牢固树立本领恐慌意识，不断加强学习，打牢干事创业的能力基础。二要坚定信念。牢固树立发展自信、改革自信、创新自信，真正做到改革不为风险所惧、干事不为名利所累、前行不为干扰所惑。三要深入调研。既要吃透“上情”，准确把握上级精神的实质内涵，又要摸准“下情”，接通地气掌握基层一线实情，富有成效地开展工作。四要民主决策。认真落实民主集中制，尤其是对涉及经济社会发展的重大项目、重大工程和重大事项，要认真进行专家论证、风险评估和合法性审查，广泛听取社会各界人士的意见和建议，确保政府决策更加有效可行、更加符合实际。

要坚持依法行政。一要自觉接受人大及其常委会的法律监督，主动接受政协的民主监督，认真办理人大代表意见、建议、议案和政协委员提案。支持工会、共青团、妇联等群团组织开展工作。二要牢固树立法治思维和法治意识，严格按照法定权限和程序行使权力、履行职责，不断提高依法行政水平。三要推行“阳光”政务，拓宽政府信息公开渠道，重视发挥新闻媒体和社会公众的监督作用，保证人民群众的知情权、参与权、监督权。同时，完善行政复议制度，加大行政不作为、乱作为现象整治力度，切实推动政府工作向制度化、规范化、程序化、法治化方向迈进。

要坚持务实勤政。一要敢于担当。创业维艰，奋斗以成。面对困难和挑战，政府各级干部都要责无旁贷地担当起实施“五大”战略、建设城郊强区的重任，真正做到担任不误、担难不却、担险不惧、担屈不计，以无愧于历史的业绩，回馈人民的信任和支持。二要转变作风。严格执行中央和省、市关于改进工作作风的各项规定，全面加大“庸、懒、散、奢”等机关病治理力度，切实解决好“门难进、脸难看、话难听、事难办”的问题。三要真抓实干。对于区人代会报告确定的各项目标任务，各乡镇政府和区直各部门都要认真研究推进措施，逐项明确责任主体，制定细化具体要求，切实做到年初有安排、年中有督查、年底有交待，向人民群众交上一份满意的答卷。

要坚持廉洁从政。廉洁是立身之本、成事之基、执政之要。政府各级干部都要常修从政之德，常怀律己之心，坚持自重、自省、自警、自励，筑牢思想道德防线，不碰法律法规红线。一要严格执行中央“八项规定”，加强“四风”问题整治，切实推动干部作风形象的根本好转。二要勤俭办事，认真抓好增收节支，合理确定部门预算，严格预算支出的审批和管理，确保“三公”经费逐年递减，行政成本继续降低。三要扎实推进廉政风险防控体系建设，建立健全建筑工程、土地交易、政府采购、资金监管等重点领域的反腐倡廉长效机制，真正以廉洁高效的形象取信于民。

各位代表，使命在身，重任在肩。面对新形势和新任务，人民在期待着我们，重托在激励着我们。让我们在区委的正确领导下，紧紧依靠全区人民，咬定目标，只争朝夕，开拓创新，锐意进取，为打造生态宜居新城、建设现代城郊强区而努力奋斗！
